# Self-perceived competence correlates poorly with objectively measured competence in Evidence Based Medicine among medical students

**DOI:** 10.1186/1472-6920-11-25

**Published:** 2011-05-28

**Authors:** Nai Ming Lai, Cheong Lieng Teng

**Affiliations:** 1 Department of Paediatrics, Monash University Sunway Campus, Jeffrey Cheah School of Medicine and Health Sciences, JKR 1235, Bukit Azah, 80100, Johor Bahru, Johor, Malaysia; 2 Department of Family Medicine, International Medical University, Jalan Rasah, 70000, Seremban, Negeri Sembilan, Malaysia

**Keywords:** Evidence Based Medicine, assessment, undergraduate

## Abstract

**Background:**

Previous studies report various degrees of agreement between self-perceived competence and objectively measured competence in medical students. There is still a paucity of evidence on how the two correlate in the field of Evidence Based Medicine (EBM). We undertook a cross-sectional study to evaluate the self-perceived competence in EBM of senior medical students in Malaysia, and assessed its correlation to their objectively measured competence in EBM.

**Methods:**

We recruited a group of medical students in their final six months of training between March and August 2006. The students were receiving a clinically-integrated EBM training program within their curriculum. We evaluated the students' self-perceived competence in two EBM domains ("searching for evidence" and "appraising the evidence") by piloting a questionnaire containing 16 relevant items, and objectively assessed their competence in EBM using an adapted version of the Fresno test, a validated tool. We correlated the matching components between our questionnaire and the Fresno test using Pearson's product-moment correlation.

**Results:**

Forty-five out of 72 students in the cohort (62.5%) participated by completing the questionnaire and the adapted Fresno test concurrently. In general, our students perceived themselves as moderately competent in most items of the questionnaire. They rated themselves on average 6.34 out of 10 (63.4%) in "searching" and 44.41 out of 57 (77.9%) in "appraising". They scored on average 26.15 out of 60 (43.6%) in the "searching" domain and 57.02 out of 116 (49.2%) in the "appraising" domain in the Fresno test. The correlations between the students' self-rating and their performance in the Fresno test were poor in both the "searching" domain (r = 0.13, p = 0.4) and the "appraising" domain (r = 0.24, p = 0.1).

**Conclusions:**

This study provides supporting evidence that at the undergraduate level, self-perceived competence in EBM, as measured using our questionnaire, does not correlate well with objectively assessed EBM competence measured using the adapted Fresno test.

**Study registration:**

International Medical University, Malaysia, research ID: IMU 110/06

## Background

Evidence Based Medicine (EBM) has been incorporated into the curricula of many medical schools over the past two decades. Teaching learning activities and assessments in EBM are mainly based on the clearly defined domains of asking answerable clinical questions, searching for evidence, appraising the evidence and applying the evidence [1]. Competence in EBM, either self-perceived or objectively measured, has been assessed extensively. Numerous tools have been developed for this, encompassing part or all of the EBM domains, and some have been validated more comprehensively than others [2,3].

Evaluating self-perceived competence has its merits, as it may provide an indication on the subject's motivation in maintaining and improving the skills concerned, as self-perceived competence is proposed as one component in the concept of self-efficacy [4]. However, objective assessment tools are often regarded as the gold standards in measuring competence, and studies on physicians found that their self-assessment of clinical skills did not correlate well with external assessment of the same skills, and the most inaccurate self-assessments were observed in the physicians who expressed the highest confidence or those who were externally-rated to be the lowest [5]. On the other hand, studies conducted on either medical students or junior doctors showed more variable results in terms of correlation between self-perceived and objectively measured or observed competence, with poorer correlations in practical clinical skills, and comparatively better correlations in the "soft" skills like communication skills [6-11].

The practice of EBM involves the use of a mixture of "soft" skills (like critical thinking in identifying clinical questions, determining the clinical circumstances and applicability of the findings to the patients) and technical skills (like searching online resources and deriving and interpreting statistics)[1], and to-date, there is still a paucity of evidence on the extent of association between self-perceived competence and objectively measured competence in EBM. Two earlier studies at postgraduate level showed weak association between self-perceived competence and objectively measured competence in EBM, and demonstrated that the objectively measured competence of the participants was lower than expected [12,13]. One similar study has been conducted on the undergraduates, using a web-based assessment tool to assess self-reported competence and actual competence through a series of multiple choice questions that evaluated understanding and application of certain key concepts in EBM [14]. The study also showed poor association between the students' self-perceived competence and objectively assessed competence. However, the aforementioned three studies used assessment instruments that focused on one specific area in EBM, either in literature appraisal [13] or in the understanding on the EBM terms [12,14], and they were conducted before the introduction of validated instruments that assess a wide range of EBM related skills, which have only been developed relatively recently [2].

At International Medical University, Malaysia, EBM is incorporated into the medical curriculum throughout its five-year medical training program. The students are exposed progressively to EBM since their pre-clinical phase in the first two-and-a-half years, through lectures, problem-based learning, research projects and short EBM summaries, culminating in a clinically-integrated EBM training program in the final six-months of their medical training, the senior clerkship. The EBM training program in senior clerkship is elaborated in our Methods. In 2006, we conducted a pre-and-post study to assess the competence in EBM of our final-year medical students at the beginning and the end of senior clerkship using a validated assessment instrument, the Fresno test [15]. During the post-test, we also piloted a survey questionnaire that evaluated the students' self-perceived competence in EBM and their perceptions on the value and barriers of EBM in clinical practice. We hoped the findings of this survey would provide some indications on the students' self-efficacy in EBM related skills and their readiness to practice EBM, which in turn would serve as supporting evidence, alongside their objectively measured EBM competence, on the strength and deficiencies of our EBM training program in senior clerkship. Both the Fresno test and our questionnaire contained items that assess similar domains in EBM, enabling a comparison between self-perceived and objectively measured competence in EBM.

In this paper, we report the students' self-perceived competence in EBM as measured by our pilot questionnaire, and the correlation between this and their objectively measured competence in EBM, as represented by the students' performances in the Fresno test. We selected matching items from both tools for correlation, as detailed in our Methods. We set the following research question: Were there significant and important correlations between our medical students' self-perceived competence and their objectively measured competence in the various domains of EBM? We defined significant correlations as the correlations that were statistically significant (as represented in this study by p values of less than 0.05), and important correlations by the correlation coefficients of at least 0.5.

## Methods

### Study design

This was a cross-sectional study. This study was part of a project that assessed student competence in EBM following a clinically-integrated EBM training program. Part of the data that we use for this study, the objectively-measured EBM competence (the "post-test" scores in the adapted Fresno test), has been published previously [15].

### Participants

Our participants comprised of a group of medical students (n = 72) from the International Medical University of Malaysia. The students were in their final six months of training ("senior clerkship"), from March to August 2006.

### EBM training in senior clerkship

The students received a structured, clinically-integrated EBM training program within their six-month senior clerkship. This was the final phase of their undergraduate EBM training. This EBM training program, first developed in May 2003, consisted of overview lectures, searching and critical appraisal, and small-group training integrated with bedside clinical sessions and journal club, in which students critically appraised clinical articles and undertook exercises in deriving and interpreting common statistical expressions like relative risk, absolute and relative risk reduction, number needed to treat (NNT) and the likelihood ratio. Throughout the training program, the students were expected to demonstrate an ability in formulating relevant answerable clinical questions, performing searches using appropriate search strategies and identifying the best study type that matches their clinical queries, appraising evidence retrieved from their searches and in the process understanding basic statistical expressions that were commonly reported in the clinical papers, and determining the applicability of the appraised evidence to the local population. Each student was required to develop five EBM reports across different disciplines during their six-month clerkship period using the aforementioned skills. These EBM reports constituted parts of the documents on which the students would be assessed summatively via an oral examination at the end of their medical training. The training program was jointly developed by both authors (NML and CLT). All training sessions including the introductory lectures and small-group sessions were facilitated by the first author (NML), who was then the coordinator of senior clerkship program. The current study was conducted at the end of the students' EBM training in senior clerkship in July 2006.

### Outcome measures

#### i). Self-perceived competence in EBM - pilot questionnaire

This was one of the three major components evaluated in our survey. The other two components were i) Attitude: perceived value of EBM in clinical practice (five items), and ii). Perceived barriers to practicing EBM (four items). As this paper focuses on the correlation between self-perceived competence and objectively measured competence, we report only the component of self-perceived competence.

There were 16 items in our questionnaire dedicated to measuring self-perceived competence in EBM. The 16 items were grouped under the following components:

i). Estimated speed in online search (Question one: with a five-point rating scale)

ii) Satisfaction with search results (Question two: with a five-point rating scale)

iii). Frequency in which the respondents could tell a good study from a not-so-good study (Question three: with a five-point rating scale)

iv). Understanding of different sections of an article (Questions four to seven: each with a four-point rating scale)

v). Ability to perform critical appraisal (Question eight: with a four-point rating scale)

vi). Understanding on EBM terms (Questions nine to 16: each with a four-point rating scale).

The questionnaire was adapted from the Student Competency Survey Questionnaire developed in November 2004 to measure our students' self-perceived competence across a range of clinical, practical and personal skills. Studies using various components of the questionnaire have been published [16-18]. We developed the current questionnaire by expanding the five-item relating to students' information-seeking practice in the Student Competency Survey Questionnaire [18]. Specifically, we added components ii, iii and vi, and removed two components relating to the preferred sources of information and the frequencies in accessing different informational sources, as these were not considered directly relevant in assessing self-perceived competence. Additionally, in component iv, we expanded the single item on the overall understanding of a journal article into four items, each dealing with the understanding on different parts of a paper, i.e. introduction, methods, results and conclusion. In component vi, we chose eight common EBM glossary terms based on the questionnaire developed by McColl et al [19].

The face validity of the questionnaire was assessed by a panel of three experienced teachers of EBM that included both authors. The previous five-item version of the questionnaire showed good internal consistency [18]. This is the pilot for the current expanded version.

#### ii). Objectively measured EBM competence

We measured this using the adapted Fresno test of competence in EBM (score: 0 to 212). We made some adaptations to the original version developed and validated by Ramos et al [20] to tailor to our undergraduates, and piloted the revised version on a group of 12 medical students. The details of our adaptations are reported in a separate paper [15]. Both authors independently scored the completed test scripts, guided by a grading rubric. We then analysed our differences in the final scores and obtained a mean difference and standard deviation. If the difference between our final scores was more than two standard deviations apart, we discussed the scripts concerned question-by-question, leading to a consensus score. For all other scripts, we averaged the final scores.

### Matching items in the questionnaire and the Fresno test

We categorised the items in both tools under the four major domains of EBM. Table 1 displays the results of our categorization. We noted that while the Fresno test covered all four domains, our questionnaire focused on two: "searching for evidence" and "appraising the evidence" (Table 1). Within the same domain, there were related items in both instruments which would enable meaningful correlations. For example, under the domain of "searching for evidence", question two in Fresno test required the candidates to list the possible sources for searching clinical evidence, and describe the strengths and limitations of each source listed, and question four required the candidates to describe their search strategy for the clinical scenario in question one. Under the same domain in our questionnaire, items one and two covered the estimated speed and satisfaction of search respectively. Under the domain of "Appraising the evidence", the items in the Fresno test covered the assessment of knowledge on study design, internal validity, clinical importance including the derivation and interpretation of EBM expressions such as absolute and relative risk reduction and likelihood ratio. The corresponding items in the questionnaire, on the other hand, covered the understanding of an article on the whole, the self-perceived ability to perform critical appraisal and the understanding of common EBM terms such as those assessed in the Fresno test. Our consensus was that since the items under different domains appeared to evaluate separate constructs, we would not perform an overall correlation between the two instruments. Instead, we would correlate only items under the same domain.

**Table 1:** BM domains evaluated by the Fresno test and our questionnaire

Question number		Domains evaluated
	
Fresno test	Questionnaire	
1		Asking question

2,4	1,2	Searching for the evidence

3,5,6,8 to 12	3 to 16	Appraising the evidence

7		Applying the evidence

### Conduct of the study

We invited our students to participate in the study by completing our questionnaire and the adapted Fresno test concurrently. Prior to the study, the students received a study information leaflet and a briefing from the first author. In the briefing, the students were informed that participation was voluntary, and their decision to participate or not would not affect their university standing. We obtained written consent from students who agreed to participate. An administrative staff member oversaw the consent signing in the absence of any investigator. All students received the EBM training, a standard program in the university senior clerkship curriculum, whether or not they participated.

### Statistical analyses

We combined the students' ratings of all the items under the same EBM domains to form a sum rating in their self-perceived competence. For example, we combined ratings of questions one and two to form a sum rating in "searching for the evidence" and likewise for "appraising the evidence". We also performed the same with the items in the adapted Fresno test, and obtained a sum score under a specific domain in the Fresno test. We correlated the sum rating of a domain in our questionnaire with the sum score of the same domain in the Fresno test. We assessed all correlations using Pearson's product-moment correlation (PASW 18 (Chicago, IL, USA)).

### Post-hoc power analysis

We performed a post-hoc power analysis after completing our study, using the methods of Faul et al via the G*Power software [21]. We considered a correlation coefficient (r) of at least 0.5 to be important, and set a correlation coefficient of zero as a reference for our null hypothesis with alpha of 0.05. We accepted a power of at least 80%. Our sample of 45 participants provided a power of 94.9% in detecting such a degree of correlation.

### Research and Ethics Committees approval

The study was approved by the Research and Ethics Committees, International Medical University, Malaysia.

## Results

Forty five out of 72 students (62.5%) participated by completing both our questionnaire and the Fresno test. The internal consistency of the 16 items that evaluated self-perceived competence in our pilot questionnaire, measured using Cronbach's alpha, was 0.79 (95% confidence interval for intra-class correlation coefficient: 0.70 to 0.86). All items except question one ("estimated time in tracing an abstract of interest") contributed positively to the internal consistency of the questionnaire. After deleting question one, the internal consistency improved to 0.80. We decided to retain question one in this report for its practical relevance. For the adapted Fresno test, inter-rater correlation was 0.90 (95% CI: 0.87-0.93). Excluding question 12, a multiple choice question, inter-rater correlation for individual questions ranged from 0.52 to 0.98. The scores from two raters differed by an average of 13.1 points (6.2%) (SD 13.0 (6.1%)). The average Fresno test score among the 45 students who participated in this study was 119.2 (56.2%) (SD 21.5 (10.1%)).

The survey responses of the students are displayed in Table 2 (for questions one to three) and Table 3 (for questions four to 16). From Table 2, half of our students (50.1%) reported that they took less than 30 minutes to trace an abstract of interest. Only a minority (17.8%) were satisfied with their search results at least "majority of the time". None of the participants reported that they were able to tell a good study from a not-so-good study either "often" or "all or most of the time" in their reading.

**Table 2:** Self-perceived competence in EBM: students' responses shown in proportions for the first three questions

Question			
1. Estimated time in tracing an abstract of interest	" > 1 hour or mostly not traceable"	"Between 30 minutes to 1 hour"	"< 30 minutes"
	
	8(17.8)	14(31.1)	33(51.1)

2. Satisfaction with search results	"Less than half of the time or very seldom/never"	"Around half of the time"	"Majority, most or all of the time"
	
	16(35.5)	21(46.7)	8(17.8)

3. Ability to tell a good study from a not-so-good study	"Occasionally to very rarely or never"	"Sometimes"	"Often, most or all of the time"
	
	25(58.1)	18(41.9)	0

We simplified the presentation by merging ratings one and two (e.g. "mostly not traceable" and "> 1 hour" were combined into "> 1 hour or mostly not traceable") as well as ratings four and five (e.g. "< 10 minutes" and "between 10 and 30 minutes" were combined into "< 30 minutes") for each question.

**Table 3:** Self-perceived competence in EBM: students' responses shown in proportions for the questions four to sixteen, each with four response ratings

Question	Frequency of response (percentage)			
Understanding of an article: 4. Introduction	"Not at all"	" Partially"	"Sufficiently but not fully"	"Fully"
	
	0	0	18(40)	27(60)

5. Methods	0	5(11.1)	35(77.8)	5(11.1)

6. Results	0	8(17.8)	31(68.9)	6(13.3)

7. Conclusion	0	0	22(48.9)	23(51.1)

8. Ability to perform critical appraisal	"Have not a clue about critical appraisal"	"Need a lot of guidance in appraising all types of study"	"Confident in appraising only certain types of study"	"Confident in appraising all common types of study"
	
	0	2(4.5)	36(81.8)	6(13.6)

Understanding on EBM glossaries 9. Sensitivity/Specificity	"Unaware"	"Heard about it"	"Understand"	"Can explain"
	
	0	2(4.4)	27(60.0)	16(35.6)

10. Predictive values	0	5(11.1)	29(64.4)	11(24.4)

11. Relative risk/Odds ratio	0	2(4.4)	34(75.6)	9(20.0)

12. Absolute risk reduction	1(2.2)	14(31.1)	24(53.3)	6(13.3)

13. Number needed to treat (NNT)	3(6.7)	19(42.2)	19(42.2)	4(8.9)

14. Randomisation	0	2(4.4)	21(46.7)	22(48.9)

15. Blinding	0	2(4.4)	21(46.7)	22(48.9)

16. Meta-analysis	0	6(13.3)	26(57.8)	13(28.9)

From Table 3, all students reported that they understood the introduction and the conclusion of an article either sufficiently or fully. Comparatively, they appeared less confident in reading the methods and the results of an article, as small proportions reported only partial understanding of the two sections (11.1% for methods and 17.8% for results). However, almost all students (95.5%) reported that they were confident in appraising at least certain types of study. The vast majority (over 85%) indicated that they were familiar with six out of eight EBM terms by choosing "understand" or "can explain" for those terms. The remaining two terms, "absolute risk reduction" and "number needed to treat" appeared to be the most difficult, with over 30% reported that they were either unaware of the terms or had only heard about them.

Table 4 shows the students' self-perceived competence, in the form of their sum-ratings in the questionnaire, together with their objectively measured competence, in the form of their sum-scores in the Fresno test under the two domains assessed. Comparisons between the students' self-perceived competence in EBM, as measured by our questionnaire, and their scores in the Fresno test were only possible in two domains ("searching for the evidence" and "appraising the evidence") because our questionnaire contained no item in the other two domains ("asking questions" and "applying the evidence"). Comparing the students' sum-ratings and their sum-scores in percentages, the students in general appeared to rate themselves higher than their actual performances.

**Table 4:** Sum-ratings (questionnaire) and sum-scores (Fresno test) in the two EBM domains covered in both instruments

Domain	Tool	Mean sum rating/sum score	Standard deviation	Maximum sum rating/sum score	Mean sum rating/sum score in percentage
Searching for the evidence	Questionnaire	6.34	1.67	10	63.4%
	
	Fresno test	26.15	6.82	60	43.6%

Appraising the evidence	Questionnaire	44.41	4.46	57	77.9%
	
	Fresno test	57.02	16.03	116	49.2%

The correlation coefficient (r) between the students' self-rating and their Fresno test scores in the domain of "searching for evidence" was 0.13 (p = 0.4). In the domain of "appraising the evidence", the correlation coefficient between the self-rating and test performances was 0.24 (p = 0.1).

## Discussion

Our pilot questionnaire demonstrated good overall internal consistency among the 16 items that assesses self-perceived competence in EBM. It shows that in most items, our students rated themselves as moderately competent in EBM. However, our students' self-perceived competence in EBM correlated poorly with their performances in the adapted Fresno test in both the domains of "searching for evidence" and "appraising the evidence".

We presented the correlation between the questionnaire responses and the Fresno test scores instead of reporting this study as a validation of our questionnaire against the Fresno test for two major reasons. First, the Fresno test, although validated, has not been widely agreed as a reference standard for measuring competence in EBM. Second, it was unclear what the essential criteria or the appropriate pass marks for each question in the Fresno test should be at the undergraduate level to convert the scoring into "pass-fail" category and enable a valid comparison with any other assessment scale.

Our principal finding confirms the findings of previous studies on EBM, and suggests that because of its poor correlation with a validated objective assessment tool, self-ratings should not be used as a stand-alone tool to evaluate undergraduate competence in EBM. Extrapolating from the findings of the systematic review by Davis et al [5] which showed that physicians who were rated the lowest in skills had the most inaccurate self-assessments, our findings of poor correlations between the medical students' self-perceived competence and objectively assessed competence was not surprising, as the medical students were very likely to receive low external ratings in terms of skills using the same standards for judging a physician. Nonetheless, our study provided some interesting insights in terms of EBM training at the undergraduate level. At the completion of their undergraduate EBM training, our students managed to attain a moderate level of self-perceived competence, but this level of competence was not consistent with the objective measurement using the adapted Fresno test. The students' self-ratings suggested that they might have acquired some skills in EBM but found it difficult to apply their skills in practice. For instance, while the vast majority reported that they were confident in critically appraising at least certain types of studies, they appeared less confident in understanding the methods and the results of an article, and few were able to consistently tell a good study from a not-so-good study, which requires the application of critical appraisal skills. Similar discrepancy was observed in searching, as the majority indicated that they took less than 30 minutes to trace an abstract of interest, while only a minority was often happy with their search results. A previous study on our students shows that the majority performed their searches either in the primary databases like PubMed or in single journals [18]. This might result in either overly broad or overly narrow search yields, which might lead to frustration if the initial article of interest turned out to be unsuitable. This suggests that future EBM training should emphasize on how to search efficiently by focusing on the appropriate clinical information sources. Our findings collectively also imply a need to critically evaluate our EBM training program to determine the appropriate amount of EBM skills in each domain to be imparted to our students, taking into account of their relevance and retainability, and to highlight to the students the preparatory nature of their undergraduate EBM training and set realistic targets on their expected competence.

### Limitations

An obvious limitation of our study is that we performed correlation between a pilot questionnaire and an adapted version of a fully validated assessment tool. Although the current version of our questionnaire has undergone an assessment for its face validity and demonstrated good internal consistency, the result of our pilot suggests a need to further revise the questionnaire, as one of the items, "estimated time in tracing an abstract of interest" had a negative contribution to the overall internal consistency. Also, our questionnaire items do not appear to cover EBM domains in equal breadth and depth as the Fresno test. Comparing the items under both "searching for evidence" and "appraising the evidence", most items in our questionnaire were more superficial than the corresponding items in the Fresno test. Although the difference in the level assessed does not preclude an exploratory correlation study like what we have undertaken here, caution is needed in interpreting the results, as the corresponding items between the two scales might not have been sufficiently matched. Next, it is worth noting that the original Fresno test was validated a group of practicing health care staff and not on medical undergraduates. Our adaptation for the undergraduates, despite having gone through a round of piloting, could have been further improved.

## Conclusions

Our study provides supporting evidence that medical students' self-perceived competence in EBM measured using our questionnaire correlated poorly with objectively assessed EBM competence measured using the adapted Fresno test. We propose that self-perceived competence is not a reliable measure of student competence in EBM, and it should not be used solely to indicate the effectiveness of an EBM training program at the undergraduate level. Further research correlating self-perceived competence with objectively measured competence in EBM should be conducted using two instruments that are validated and contain sufficiently-matched items under various EBM domains.

## Competing interests

Both authors declare to have no financial or non-financial interests that may be relevant to the submitted work.

## Authors' contributions

NML conceived the study, wrote the research protocol, developed the questionnaire, adapted the Fresno test and the grading rubrics, recruited the subjects, scored the Fresno test, analysed the data, and wrote the manuscript. CLT developed the questionnaire, participated in adapting the test and grading rubrics, scored the Fresno test, and assisted in writing the manuscript. Both authors have read and approved the final manuscript. Guarantor: NML

## Authors' information

NML is a Senior Lecturer in Paediatrics at the Monash University Sunway Campus, Malaysia. He is involved in delivering teaching and assessing students in the Evidence Based Clinical Practice (EBCP) module in years three and four. He was formerly from International Medical University, Malaysia. CLT is a Professor of Family Practice and the main coordinator of Evidence Based Medicine curriculum at the International Medical University, Malaysia.

## Pre-publication history

The pre-publication history for this paper can be accessed here:

http://www.biomedcentral.com/1472-6920/11/25/prepub
